# Supplementary material for: Investigating the role of long non-coding RNA in hypertrophic cardiomyopathy
Source: bioRxiv. 2025 Jul 31:2025.07.26.666851. Preprint. [Version 1] doi: 10.1101/2025.07.26.666851 (PMC12324334; doi:10.1101/2025.07.26.666851)
Supplement: Supplement 1 [file media-1.zip › lncrna_hcm/supplementary_material/supp_table_1.pdf]

Supplemental Table 1: lncRNA genes that are DE in mouse.

Mouse (GRCm38/mm10)					Human (GRCh38/hg19)			
**lncRNA gene name (Ensembl ID)**	**comparison source**	**log$_2$FC**	***p*-adj**	**known tissue expression**	**lncRNA ortholog gene name (Ensembl ID)**	**FC info**	**known tissue expression**	**known function**
Gm43672 (ENSMUSG00000106019)	TG vs nTG	-0.557	7.62E-11	Heart, brain, muscle, trachea, skin, spleen<br>https://rnacentral.org/rna/URS0002A33156/10090	ENSG00000308496			
	TG_f vs nTG_f	-0.630	1.04E-06					
	TG_HIIT vs nTG_HIIT	-0.583	3.17E-06					
	TG_Sed vs nTG_Sed	-0.531	6.55E-04					
	TG_m vs nTG_m	-0.486	1.99E-03					
Gm9905 (ENSMUSG00000053358)	TG vs nTG	-0.594	3.10E-08					
	TG_HIIT vs nTG_HIIT	-0.689	3.17E-06					
	TG_m vs nTG_m	-0.587	1.74E-03					
	TG_f vs nTG_f	-0.602	2.28E-03					
Gm20632 (ENSMUSG00000093577)	TG_HIIT vs nTG_HIIT	-0.546	1.80E-03	All tissues have low expression<br>https://rnacentral.org/rna/URS0002A237A7/10090	ENSG00000290792			
Gm42918 (ENSMUSG00000106209)	TG_m vs nTG_m	-0.951	9.07E-04					
	TG vs nTG	-0.680	1.20E-03					
	TG_HIIT vs nTG_HIIT	-0.810	4.42E-03					
Banf2os (ENSMUSG00000086384)	TG vs nTG	-0.965	3.11E-08	All tissues have low expression<br>https://rnacentral.org/rna/URS0002A19B47/10090				
	TG_f vs nTG_f	-1.086	1.38E-04					
	TG_Sed vs nTG_Sed	-1.047	6.55E-04					
	TG_HIIT vs nTG_HIIT	-0.883	1.80E-03					
	TG_m vs nTG_m	-0.842	1.01E-02					
Gm13375 (ENSMUSG00000075514)	TG_HIIT vs nTG_HIIT	-0.488	4.46E-03	Brain, skin, bone, thymus, lung, colon, spleen<br>https://rnacentral.org/rna/URS0002A2504A/10090				
RP23-166L22.2 (ENSMUSG00000110340)	TG vs nTG	-1.361	1.64E-06	All tissues have low expression				
	TG_f vs nTG_f	-1.740	5.94E-03					

	TG_m vs nTG_m	-1.159	1.01E-02					
G730003C15Rik (ENSMUSG00000097573)	TG_HIIT vs nTG_HIIT<br>TG vs nTG	-0.684<br>-0.536	5.47E-03<br>8.45E-03	https://rnacentral.org/rna/URS0002A4F318/10090	ENSG00000287133			
E230013L22Rik (ENSMUSG00000096957)	TG vs nTG<br>TG_HIIT vs nTG_HIIT<br>TG_f vs nTG_f	-0.476<br>-0.539<br>-0.525	9.14E-05<br>1.80E-03<br>2.13E-02	Heart<br><br>https://rnacentral.org/rna/URS0002A1C2B5/10090				Most upregulated lncRNA after X-ray irradiation in mouse lung tissue, modulates the mRNA BNIP1[44]
2900072N19Rik (ENSMUSG00000087018)	TG vs nTG	-0.760	8.45E-03	Brain<br><br>https://rnacentral.org/rna/URS0002A5782B/10090	LINC02710 (ENSG00000255269)		Testis<br><br>https://rnacentral.org/rna/URS0000EF618F/9606	
C530005A16Rik (ENSMUSG00000085408)	TG vs nTG	-0.414	9.85E-03	Brain<br><br>https://rnacentral.org/rna/URS0002A5A50C/10090				
Prr33 (ENSMUSG00000043795)	TG_HIIT vs nTG_HIIT<br>TG_m vs nTG_m<br>TG vs nTG	-0.519<br>-0.499<br>-0.373	1.80E-03<br>1.01E-02<br>2.68E-02					Annotated in GENCODE M10 as long intergenic non-coding RNA, but traditionally annotated as protein-coding.
Gm37752 (ENSMUSG00000103385)	TG vs nTG	2.438	1.83E-02					
Gm45012 (ENSMUSG00000109052)	TG vs nTG<br>TG_HIIT vs nTG_HIIT	-0.422<br>-0.415	9.49E-04<br>4.00E-02	Heart<br><br>https://rnacentral.org/rna/URS0002A2D746/10090	ENSG00000300258			
PARTICL (ENSG00000286532)	TG_HIIT vs nTG_HIIT	-0.468	2.45E-02	Heart, arteries, prostate, esophagus, brain, fallopian tubes, cervix, testis, colon, thyroid, bladder, skin<br><br>https://rnacentral.org/rna/URS0002A47137/9606	PARTICL (ENSG00000286532)			Upregulated following low-dose irradiation. Alters H3K27me3 distribution and interacts with DNA methyltransferase 1.[45,46] Forms triple helices clustered at tumor suppressor genes.[47]
Mir223hg (ENSMUSG00000078122)	TG_m vs nTG_m<br>TG_HIIT vs nTG_HIIT	0.685<br>0.520	1.99E-03<br>4.72E-02	Heart, lung, Spleen, liver, skin, mammary gland, bone, uterus,	Mir223hg (ENSG00000274536)		Appendix, tonsil, lung, bladder, bone, leukocyte, lymph node,	Positive regulator of inflammation in atherosclerosis.[57,58] Differentially expressed in lung adenocarcinoma and ovarian cancer.[48,49]

				bladder, adrenal gland,<br><br>https://rnacentral.org/rna/URS0002A33BA1/10090			small intestine, spleen<br><br>https://rnacentral.org/rna/URS0002A35E6A/9606	
Gm28703 (ENSMUSG00000099512)	TG_m vs nTG_m<br>TG vs nTG	-0.616<br>-0.470	2.11E-02<br>2.89E-02	All tissues have low expression<br><br>https://rnacentral.org/rna/URS0002A2C79C/10090				
Gm10435 (ENSMUSG00000072902)	TG vs nTG<br>TG_m vs nTG_m<br>TG_HIIT vs nTG_HIIT	-0.389<br>-0.428<br>-0.387	1.93E-03<br>3.38E-02<br>4.40E-02	Heart, lung,<br><br>https://rnacentral.org/rna/URS000078648E/10090	ENSG00000287315			
9830004L10Rik (ENSMUSG00000099552)	TG vs nTG	-0.660	2.68E-02		ENSG00000300443			
Gm7628 (ENSMUSG00000025644)	TG vs nTG	-0.731	2.68E-02	All tissues have low expression<br><br>https://rnacentral.org/rna/URS0000811860/10090				
Gm17586 (A_J_v1:CM003964.1)	TG vs nTG<br>TG_HIIT vs nTG_HIIT<br>TG_m vs nTG_m	0.759<br>0.691<br>0.758	2.69E-04<br>4.40E-02<br>4.79E-02					
ANRIL (ENSG00000240498)	TG vs nTG	0.649	3.10E-02	Small intestine, colon, brain<br><br>https://rnacentral.org/rna/URS00026A21AD/9606	ANRIL (ENSG00000264545)			Transcriptional regulator and epigenetic modifier in myocardial infarction and coronary artery disease.[51] Antisense to CDKN2B. ANRIL KD has been shown to increase CDKN2B expression.[52,53] Biomarker of different caners.[50]
6330403L08Rik (ENSMUSG00000075585)	TG_HIIT vs nTG_HIIT<br>TG_HIIT vs nTG_HIIT<br>TG_HIIT vs nTG_HIIT	-0.387<br>-0.387<br>-0.387	3.14E-02<br>3.14E-02<br>3.14E-02	Heart, brain, skeletal muscle, colon, small intestine, trachea<br><br>https://rnacentral.org/rna/URS0000775964/10090	PDGFA-DT (ENSG00000223855)	HCM_m vs ctrl_m: log2FC 0.327, p-adj 2.62E-01<br><br>HCM_f vs ctrl_f: log2FC -0.499, p-adj 6.38E-01<br><br>HCM vs ctrl: log2FC 0.194, p-adj 5.17E-01	Heart<br><br>https://rnacentral.org/rna/URS000314D59/9606	

Gm45051 (ENSMUSG00000108435)	TG_HIIT vs nTG_HIIT	-0.582	3.14E-02	Kidney<br><br>https://rnacentral.org/rna/URS00009C60DF/10090				
Gm10115 (ENSMUSG00000062319)	TG_HIIT vs nTG_HIIT	0.540	3.14E-02	Heart, brain<br><br>https://rnacentral.org/rna/URS0000781B46/10090				
	TG vs nTG	0.443	3.48E-02					
Gm43318 (ENSMUSG00000105974)	TG vs nTG	1.918	3.34E-02					
Gm43267 (ENSMUSG00000104662)	TG vs nTG	-1.062	3.43E-02					
Gm43426 (ENSMUSG00000106214)	TG vs nTG	1.105	3.48E-02					
RP24-96N12.2 (ENSMUSG00000110322)	TG vs nTG	-0.335	3.48E-02	All tissues have low expression<br><br>https://rnacentral.org/rna/URS0002A60790/10090				
	TG_HIIT vs nTG_HIIT	-0.384	4.72E-02					
Gm44386 (ENSMUSG00000107689)	TG_HIIT vs nTG_HIIT	-0.540	4.40E-02	Heart, muscle, lung, adipose tissue, kidney, thymus, skin, colon, trachea<br><br>https://rnacentral.org/rna/URS0002A20769/10090	ENSG00000233060		Brain<br><br>https://rnacentral.org/rna/URS00002EFB52/9606	
Arhgap27os2 (ENSMUSG00000085360)	TG_m vs nTG_m	1.039	4.71E-02	All tissues have low expression<br><br>https://rnacentral.org/rna/URS0002A4F8BB/10090	ENSG00000294490			
RP23-299N4.1 (ENSMUSG00000109841)	TG_HIIT vs nTG_HIIT	-0.583	4.72E-02	All tissues have low expression<br><br>https://rnacentral.org/rna/URS0002A4B653/10090				
Gm17473 (ENSMUSG00000097805)	TG vs nTG	-0.884	4.94E-02	All tissues have low expression				

				https://rnacentral.org/rna/URS0002A49E0F/10090				
9430062P05Rik (ENSMUSG00000104263)	TG vs nTG	-0.467	4.95E-02					
Gm17281 (MGP_FVBNJ_G0005910)	TG_HIIT vs nTG_HIIT	-0.337	4.99E-02					

Description:

Sorted by ascending average *p*-adj value across each comparison source.

Legend:

TG: transgenic, nTG: non-transgenic, M: male, F: female, HIIT: high intensity interval training, Sed: sedentary, HCM: hypertrophic cardiomyopathy, NF: non-failing, FC: fold change, *p*-adj: Benjamini-Hochberg-adjusted *p*-value
