# Supplementary figures and images for: Investigating the role of long non-coding RNA in hypertrophic cardiomyopathy
Source: bioRxiv. 2025 Jul 31:2025.07.26.666851. Preprint. [Version 1] doi: 10.1101/2025.07.26.666851 (PMC12324334; doi:10.1101/2025.07.26.666851)

## Supplemental Fig. S1

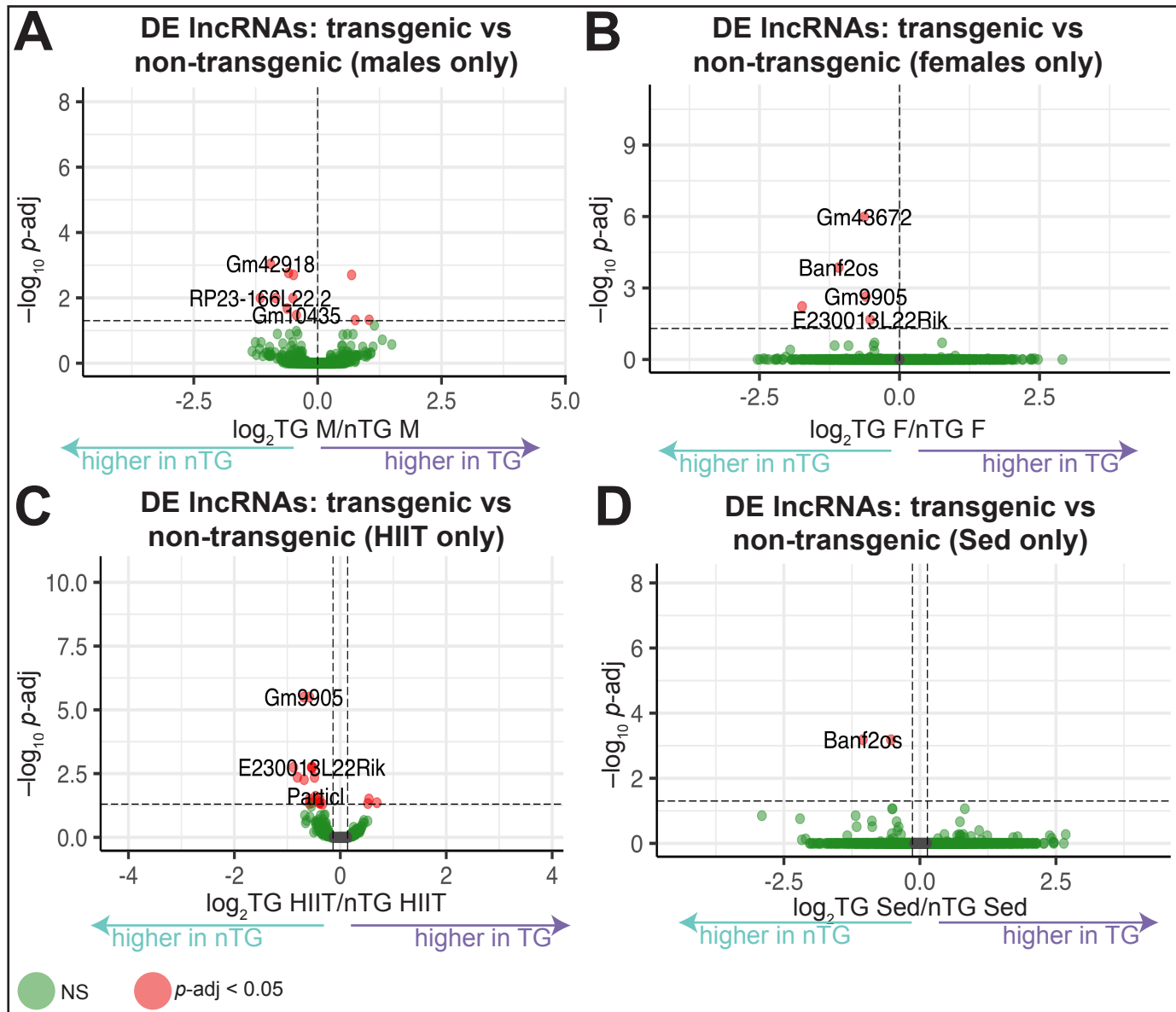

## Supplemental Fig. S2

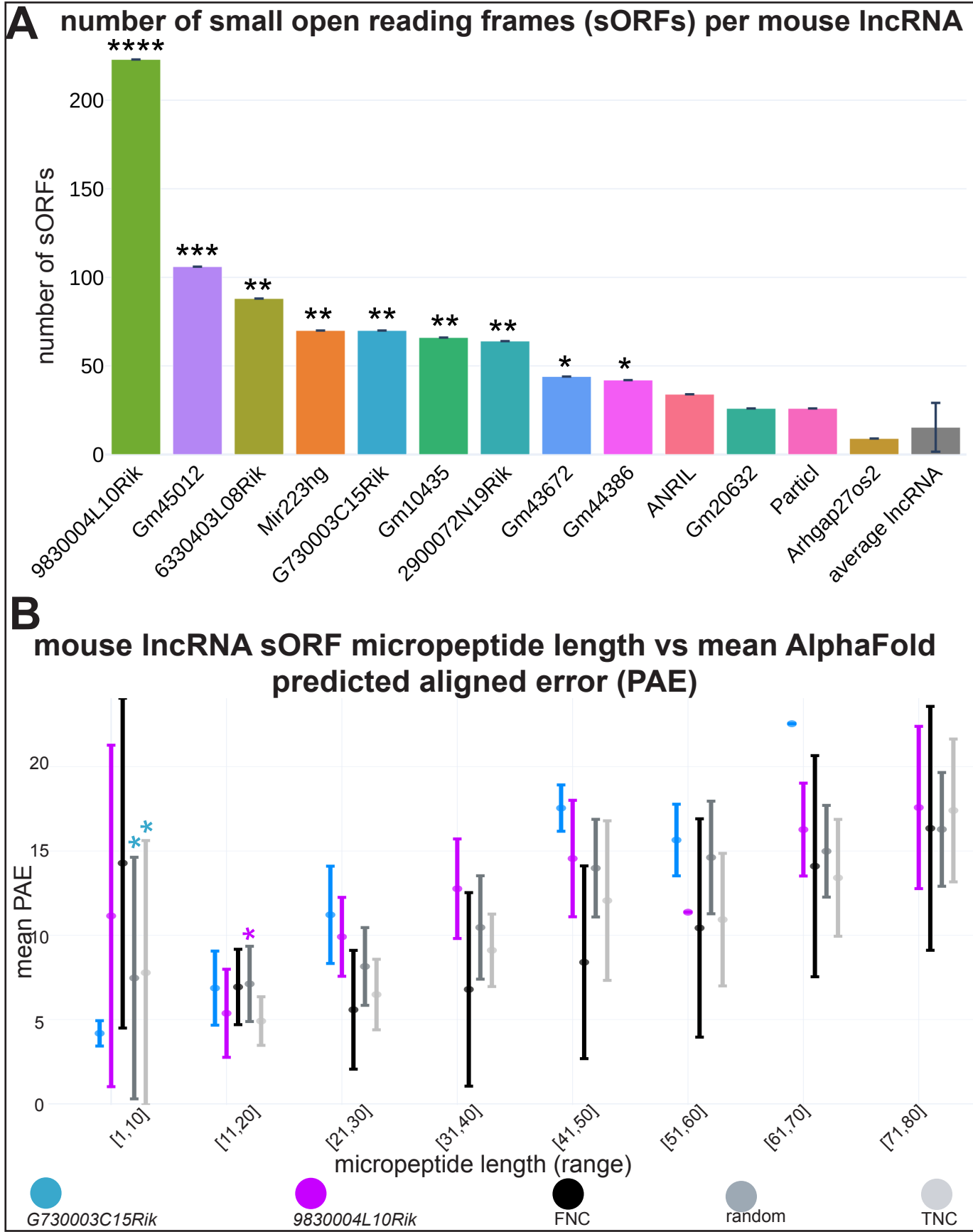

Supplemental Fig. S3

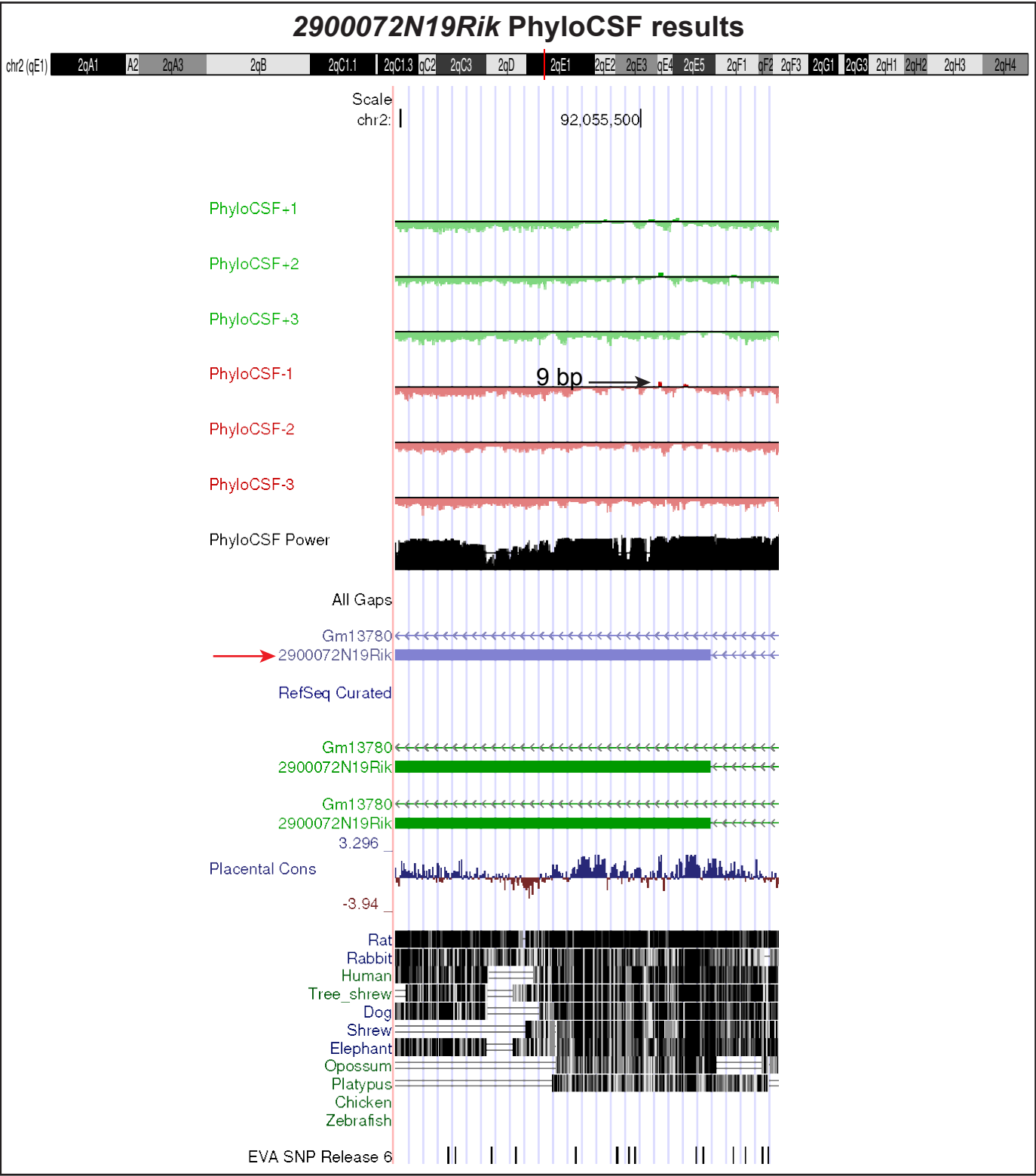

Supplement: Supplement 1 [file media-1.zip › lncrna_hcm/supplementary_material/supp_figs.pdf]
